# Quality of life in children and adolescents with growth hormone deficiency and their caregivers: an Italian survey

**DOI:** 10.1007/s40618-023-02106-3

**Published:** 2023-05-20

**Authors:** M. Maghnie, M. Orso, B. Polistena, M. Cappa, G. Pozzobon, D. d’Angela, G. Patti, F. Spandonaro, S. Granato, R. Di Virgilio, D. La Torre, M. Salerno

**Affiliations:** 1 Paediatric Clinic and Endocrinology, IRCCS Istituto Giannina Gaslini, Genoa, Italy; 2 Department of Neuroscience, Rehabilitation, Ophthalmology, Genetics, Maternal and Child Health, University of Genoa, Genoa, Italy; 3 C.R.E.A. Sanità (Centre for Applied Economic Research in Healthcare), Rome, Italy; 4 University of Rome Tor Vergata, Rome, Italy; 5 Unit of Endocrinology, Bambino Gesù Children’s Hospital, IRCCS, Rome, Italy; 6 Pediatric Clinic, IRCCS San Raffaele Hospital, Università Vita-Salute San Raffaele, Milan, Italy; 7 San Raffaele University, Rome, Italy; 8 Medical Department, Pfizer Italia, Rome, Italy; 9 Health and Value, Pfizer Italia, Rome, Italy; 10 Global Medical Affairs, Pfizer Rare Disease, Rome, Italy; 11 Department of Translational Medical Sciences, Paediatric Endocrinology Unit, University of Naples ‘Federico II’, Naples, Italy

**Keywords:** Growth hormone deficiency, Children, Quality of life, Survey, Italy

## Abstract

**Purpose:**

The aim of this study was to produce evidence on quality of life (QoL) among Italian growth hormone deficiency (GHD) children and adolescents treated with growth hormone (GH) and their parents.

**Methods:**

A survey was conducted among Italian children and adolescents aged 4–18 with a confirmed diagnosis of GHD and treated with GH therapy and their parents. The European Quality of Life 5 Dimensions 3 Level Version (EQ-5D-3L) and the Quality of Life in Short Stature Youth (QoLISSY) questionnaires were administered between May and October 2021 through the Computer-Assisted Personal Interview (CAPI) method. Results were compared with national and international reference values.

**Results:**

The survey included 142 GHD children/adolescents and their parents. The mean EQ-5D-3L score was 0.95 [standard deviation (SD) 0.09], while the mean visual analogue scale (VAS) score was 86.2 (SD 14.2); the scores are similar to those of a reference Italian population aged 18–24 of healthy subjects. As for the QoLISSY child-version, compared to the international reference values for GHD/ idiopathic short stature (ISS) patients, we found a significantly higher score for the physical domain, and lower scores for coping and treatment; compared to the specific reference values for GHD patients, our mean scores were significantly lower for all domains except the physical one. As for the parents, we found a significantly higher score for the physical domain, and a lower score for treatment; compared to reference values GHD-specific, we found lower score in the social, emotional, treatment, parental effects, and total score domains.

**Conclusions:**

Our results suggest that the generic health-related quality of life (HRQoL) in treated GHD patients is high, comparable to that of healthy people. The QoL elicited by a disease specific questionnaire is also good, and comparable with that of international reference values of GHD/ISS patients.

## Introduction

Growth hormone deficiency (GHD) is a condition caused by a lack of growth hormone secretion, and can be congenital or acquired, although in most cases its aetiology is unknown and is called idiopathic GHD [1]. Untreated GHD causes growth failure in children and adolescents and may affect skeletal mineralization, muscle strengths and lipid metabolism [2].

Growth hormone therapy is the treatment for GHD patients, accelerating the growth velocity to promote normalization of growth and stature during childhood and achieving a normal adult height, appropriate for the child’s genetic potential [3]. Health-related quality of life (HRQoL) is another important outcome in GHD children and adolescents with short stature treated with GH. HRQoL in short stature subjects defined as a height more than two standard deviations (SDs) below the mean height of a reference population matched by age and sex, has been investigated in several studies, with conflicting results [4]. The recent systematic review by Backeljauw et al. [4] found 33 studies on quality of life (QoL) in children with short stature, mostly due to GHD, idiopathic short stature (ISS), and small for gestational age (SGA); seven studies assessing the QoL in parents or caregivers of children with short stature were found.

To our knowledge, Italian studies investigating HRQoL in GHD patients are limited with one which included 80 children [5]. Thus we aimed to evaluate HRQoL in GHD patients treated with GH.

This study is part of a project comprising three phases: (1) a systematic literature review on paediatric GH treatment in Italy, having a focus on epidemiology, quality of life, treatment adherence, and economic impact, recently published [6]; (2) a cost-of-illness analysis of GHD (under preparation); (3) a survey on quality of life conducted among children and adolescents with GHD and their parents, and these results are presented in this paper.

## Methods

A survey has been conducted among children and adolescents with GHD and their caregivers, in order to investigate their HRQoL and the questionnaire was completed autonomously by the children and their caregivers. The diagnostic procedures, GH treatment and follow-up were representative of current clinical practice not requiring Ethics approval.

The aim was to analyse both the generic and disease-specific HRQoL in the overall study population and in subgroups by gender and age. In addition, our results were compared with national and international reference values.

Inclusion criteria were: (1) children and adolescents between 4 and 18 years old and their parents; (2) a confirmed diagnosis of GHD; (3) current treatment with GH.

Four clinicians with experience in GHD were involved in this study. They contributed to drafting the research protocol, in creating the questionnaire, and to inform the patients of the existence of the survey.

Data were collected between May and October 2021, using questionnaires administered through the Computer-Assisted Personal Interview (CAPI) method. Patients and their caregivers filled out the questionnaires through a web-based platform, and data were provided anonymously by masking the respondents' IP address.

The data collected included: (1) demographic data: age, gender, region of residence, Local Health Unit; (2) disease-specific data: age at diagnosis, age at signs/symptoms onset, medical specialty of the physician who made the diagnosis, medical specialty of the physician treating the patient, if the patient is managed by a centre of reference; (3) information on health resources utilization: drugs, medical examinations, diagnostic tests, day hospital and/or outpatient services, and the related direct and indirect costs; (4) quality of life of patients and caregivers, investigated through two questionnaires, one generic HRQoL, specifically the five-dimensional EuroQol questionnaire, three-level version (EQ-5D-3L), and one disease specific, the Quality of Life in Short Stature Youth (QoLISSY).

The EQ-5D-3L is a simple, very short questionnaire, validated in Italian [7] and is self-administered, to provide a measure of HRQoL. This tool is widely used in cost-effectiveness analyses; it also permits to compare QoL levels among patients with different diseases, or in healthy subjects. The questionnaire includes five items, three of which are related to functional aspects (mobility, self-care, usual activities) and the other two are related to perceived physical and mental well-being (pain or discomfort, anxiety or depression). For each item, there are three possible answers indicating the absence or presence of moderate or severe problems. The EQ-5D was completed by the children or by their parents. The algorithm proposed by Scalone et al. [8] describes the translation of the EQ-5D-3L results in quality-adjusted life years (QALYs) specific to the Italian population. The second part of the questionnaire consists of a graduated scale from 0 to 100 (Visual Analogue Scale, VAS), on which the respondent indicated his/her perceived overall health status. An additional question was included in the questionnaire, asking for the perceived level of general health (excellent/very good/good/fair/poor).

The validated Italian version of the QoLISSY questionnaire for patients and their parents [7] consists of 22 items distributed in 3 core domains, which contribute to the overall total score:

- *Physical*: refers to the physical limitations that the child can experience in everyday life due to short stature (6 items);
- *Social*: refers to how short stature interferes with the child’s social life: remarks, bullying, teasing, social isolation, feelings of rejection (8 items);
- *Emotional*: refers to the child’s feelings and emotions with regards to his short stature (being different, insecure, sad) (8 items);
- Three additional domains (28 items) cover the aspects of coping, beliefs, and treatment:
- *Coping*: refers to the way the child copes with negative feelings or experiences due to his short stature (10 items);
- *Beliefs*: refers to the child’s general beliefs about stature (4 items);
- *Treatment*: refers to the child’s experience linked to growth hormone treatment (when relevant) (14 items).
- The QoLISSY questionnaire for parents, in addition to the domains described above, consists of two further domains (16 items):
- *Future*: refers to the child’s worries about the future in relation to his short stature (5 items);
- *Effects on parents*: refers to the impact the child’s growth problem has on his parents’ feelings (helplessness, guilt, concern, anxiety, etc.) (11 items).

For each item of the questionnaires, five possible answers are available: not at all/never; slightly/seldom; moderately/quite often; very/very often; extremely/always. Items could be positively or negatively worded. Positively worded items have been scored 1–2–3–4–5 (from 1: not at all/never to 5: extremely/always) and negatively worded items have been scored 5–4–3–2–1 (from 5: not at all/never to 1: extremely/always). All sub-scale scores and the total score comprising the three core domains (physical + social + emotional) were transformed from raw score to 0–100 score, with higher values representing a higher perceived quality of life.

### Statistical analysis

Descriptive and analytical statistics were performed. Categorical variables were presented as counts and percentages, while continuous variables as means and standard deviations (SD). The results of the EQ-5D-3L questionnaire were presented for the overall population, by gender, and by gender and age group (4–7, 8–12, 13–18 years). The Pearson chi squared test was performed to investigate potential differences in the answers between gender and age groups. Two-sided Student’s *t* test was applied to compare the EQ-5D-3L utility values and the VAS score between gender and age groups.

Also the results of the QoLISSY questionnaires (child and parent versions) were presented for the overall study population, by gender, and by gender and age group (8–12 and 13–18 years for QoLISSY child; 4–7, 8–12, and 13–18 years for QoLISSY parent). The mean subscale and total scores obtained by the respondents were compared to the reference values provided in the QoLISSY Questionnaire User’s Manual [9] using the Student's t-test.

For all the statistical analyses, a p value ≤ 0.05 was considered significant.

The STATA/SE 13 software was used for all the analyses.

## Results

The overall study population included 142 children/adolescents with GHD and their parents. Patients had a mean age of 12.2 years (SD: 2.9) and were mostly males (69%). The mean age at diagnosis was 7.9 years, being lower for females (6.9 vs 8.3 years; p = 0.03), while the mean age at onset of signs/symptoms was 5.7 years, and the mean duration of treatment was 4.4 years. Most of respondents came from south/major islands and northwest, while the northeast and the central Italy were under-represented (Table 1).

**Table 1:** Characteristics of study population

	M	F	M + F
Total sample, n (%)	98 (69.0)	44 (31.0)	142
Age (years), mean (SD)	12.6 (3.0)	11.4 (2.7)	12.2 (2.9)
Age group (years), n (column %)			
4–6	5 (5.1)	3 (6.8)	8 (5.6)
7–9	9 (9.2)	7 (15.9)	16 (11.3)
10–12	28 (28.6)	20 (45.5)	48 (33.8)
13–15	41 (41.8)	12 (27.3)	53 (37.3)
16–18	15 (15.3)	2 (4.5)	17 (12.0)
Age at diagnosis (years), mean (SD)	8.3 (3.6)	6.9 (3.5)	7.9 (3.6)
Age at first signs and symptoms (years), mean (SD)	5.8 (4.1)	5.6 (3.5)	5.7 (3.9)
Years of treatment, mean (SD)	4.3 (3.2)	4.5 (3.6)	4.4 (3.3)
Geographical area, n (column %)			
Northwest	45 (45.9)	20 (45.5)	65 (45.8)
Northeast	–	2 (4.6)	2 (1.4)
Centre	4 (4.1)	–	4 (2.8)
South and major islands	49 (50.0)	22 (50.0)	71 (50.0)

### Results of EQ-5D-3L questionnaire

The EQ-5D-3L was completed by 142 respondents. As for mobility, 92% of respondents indicated they had no problems, while 8% declared to have some problems; no significant differences were found among gender and age groups, except for a higher percentage of females in the age group 13–18 years having some problems (14% vs 2% of males, p = 0.039).

Ninety-five percent of respondents had no problems with self-care. A female patient in the age group 13–17 declared to be unable to wash or dress on her own, compared to no males in the same age group (p = 0.044). Male children of 4–7 years were more likely to have problems with self-care than older males (p = 0.001).

The usual daily activities are carried out without problems by 90% of the respondents. Similarly to the self-care item, the younger males again showed more problems than the older ones (p = 0.008).

As for pain/discomfort item, 83% had no problems, while 17% experienced moderate pain/discomfort. We did not find differences between gender and age groups.

Anxiety or depression were moderately present in 10% of respondents, while two patients (1.4%) stated to be extremely anxious/depressed; 13–18-year-old females defined themselves as moderately anxious/depressed more than age-matched males (29% vs 7%; p = 0.024).

The mean (SD) VAS score was 86.2 (14.2), with no difference between males and females in the overall population and in each age group. As for the question about the perceived level of general health, 13% reported excellent health, 41% very good health, 38% good health, 8% fair health, and none reported poor health; there were no differences between gender and age groups.

The mean (SD) utility value for the EQ-5D-3L was 0.95 (0.09), with similar values between gender and age groups. The results for EQ-5D-3L are reported in Table 2.

**Table 2:** EQ-5D-3L

	M + F	M	F	M: 4–7 years	F: 4–7 years	M: 8–12 years	F: 8–12 years	M: 13–18 years	F: 13–18 years
Sample (N)	142	98	44	7	3	35	27	56	14
Mobility									
I have no problems in walking about	132 (92.3)	93 (94.9)	39 (88.6)	6 (85.7)	3 (100.0)	32 (91.4)	24 (88.9)	55 (98.2)	12 (85.7)*
I have some problems in walking about	10 (7.7)	5 (5.1)	5 (11.4)	1 (14.3)	–	3 (8.6)	3 (11.1)	1 (1.8)	2 (14.3)
I am confined to bed	–	–	–	–	–	–	–	–	–
Self-care									
I have no problems with self-care	134 (94.4)	93 (94.9)	41 (93.2)	5 (71.4) ^**#**^	3 (100.0)	32 (91.4)	25 (92.6)	56 (100.0)	13 (92.9)*
I have some problems washing or dressing myself	5 (3.5)	4 (4.1)	1 (2.3)	1 (14.3)	–	3 (8.6)	1 (3.7)	–	–
I am unable to wash or dress myself	3 (2.1)	1 (1.0)	2 (4.6)	1 (14.3)	–	–	1 (3.7)	–	1 (7.1)
Usual activities (e.g., work, study, housework, family or leisure activities)									
I have no problems with performing my usual activities	128 (90.1)	89 (90.8)	39 (88.6)	5 (71.4) ^**#**^	3 (100.0)	32 (91.4)	23 (85.2)	52 (92.9)	13 (92.9)
I have some problems with performing my usual activities	11 (7.8)	8 (8.2)	3 (6.8)	1 (14.3)	–	3 (8.6)	3 (11.1)	4 (7.1)	–
I am unable to perform my usual activities	3 (2.1)	1 (1.0)	2 (4.6)	1 (14.3)	–	–	1 (3.7)	–	1 (7.1)
Pain/discomfort									
I have no pain or discomfort	118 (83.1)	83 (84.7)	35 (79.6)	6 (85.7)	2 (66.7)	29 (82.9)	22 (81.5)	48 (85.7)	11 (78.6)
I have moderate pain or discomfort	24 (16.9)	15 (15.3)	9 (20.5)	1 (14.3)	1 (33.3)	6 (17.1)	5 (18.5)	8 (14.3)	3 (21.4)
I have extreme pain or discomfort	–	–	–	–	–	–	–	–	–
Anxiety/depression									
I am not anxious or depressed	126 (88.7)	89 (90.8)	37 (84.1)	6 (85.7)	3 (100.0)	31 (88.6)	24 (88.9)	52 (92.9)	10 (71.4)*
I am moderately anxious or depressed	14 (9.9)	8 (8.2)	6 (13.6)	1 (14.3)	–	3 (8.6)	2 (7.4)	4 (7.1)	4 (28.6)
I am extremely anxious or depressed	2 (1.4)	1 (1.0)	1 (2.3)	–	–	1 (2.9)	1 (3.7)	–	–
EQ-5D-3L utility values, mean (SD)	0.95 (0.09)	0.96 (0.08)	0.94 (0.12)	0.90 (0.19)	0.97 (0.06)	0.96 (0.07)	0.94 (0.11)	0.97 (0.05)	0.93 (0.16)
Perceived health, n (%)									
Excellent	19 (13.4)	11 (11.2)	8 (18.2)	1 (14.3)	–	2 (5.7)	7 (25.9)	8 (14.3)	1 (7.1)
Very good	58 (40.8)	40 (40.8)	18 (40.9)	1 (14.3)	1 (33.3)	15 (42.9)	9 (33.3)	24 (42.9)	8 (57.1)
Good	54 (38.0)	37 (37.8)	17 (38.6)	4 (57.1)	2 (66.7)	14 (40.0)	10 (37.0)	19 (33.9)	5 (35.7)
Fair	11 (7.7)	10 (10.2)	1 (2.3)	1 (14.3)	–	4 (11.4)	1 (3.7)	5 (8.9)	–
Poor	–	–	–	–	–	–	–	–	–
VAS Score, mean (SD) (100 = best health; 0 = worst health)	86.24 (14.19)	86.70 (13.63)	85.20 (15.49)	72.57 (19.79)	66.00 (13.89)	87.91 (13.13)	84.96 (15.98)	87.71 (12.29)	89.79 (12.05)

*Pearson Chi^2^ for gender (p < 0.05), M 13–18 vs F 13–18: Mobility (p = 0.039), Self-care (p = 0.044), Anxiety/Depression (p = 0.024)

^#^Pearson Chi^2^ for age group (p < 0.05), M 4–7 vs M 8–12/M 13–18: Self-care (p = 0.001), Usual activities (p = 0.008)

### Results of QoLISSY questionnaires

#### QoLISSY child

The QoLISSY-Child version was completed by 132 respondents aged 8–18, of which 91 males (69%) and 41 females (31%) (Table 3). The mean scores of the three core domains were all above 70/100: physical 78.66, social 74.10, and emotional 71.14, with a total score of 74.27. The additional domains obtained lower scores, in particular coping (42.65) and treatment (48.88), while the beliefs domain (68.84) was similar to the three core domains. Compared to the reference values provided in the QoLISSY User’s Manual [9], we found in our sample a significant higher score for the physical domain (78.66 vs 73.69; p = 0.0299), and lower scores for coping (42.65 vs 55.60; p < 0.0001) and treatment (48.88 vs 55.12; p = 0.0065). When considering the reference values of the QoLISSY User’s Manual [9] for GHD patients only (excluding ISS patients), the comparisons were as follows: physical (78.66 vs 80.12; p = 0.49); social (74.10 vs 80.77; p = 0.0034); emotional (71.14 vs 78.98; p = 0.0004); coping (42.65 vs 56.21; p < 0.0001); beliefs (68.84 vs 76.95; p = 0.0045); treatment (48.88 vs 54.42; p = 0.0144); total score (74.27 vs 79.96; p = 0.0048).

**Table 3:** Results of QoLISSY child

Perspective	Domains	Total (n = 132)		Male 8–12 years (n = 35)		Female 8–12 years (n = 27)		Male 13–18 years (n = 56)		Female 13–18 years (n = 14)	
		Mean (SD)	Ref. (mean) t test (p)	Mean (SD)	Ref. (mean) t test (p)	Mean (SD)	Ref. (mean) t test (p)	Mean (SD)	Ref. (mean) t test (p)	Mean (SD)	Ref. (mean) ttest (p)
Children	Physical	78.66 (20.08)	73.69 **p = 0.0299**	81.67 (16.02)	69.93 **p = 0.0001**	76.85 (22.63)	66.06 **p = 0.020**	79.69 (20.29)	80.24 p = 0.84	70.54 (22.79)	77.68 p = 0.26
	Social	74.10 (23.33)	72.94 p = 0.64	76.96 (18.84)	73.14 p = 0.24	69.79 (27.83)	64.94 p = 0.37	75.17 (24.33)	77.57 p = 0.46	70.98 (20.60)	74.26 p = 0.56
	Emotional	71.14 (19.68)	72.69 p = 0.52	72.05 (15.53)	73.08 p = 0.70	65.86 (23.24)	67.46 p = 0.72	73.88 (20.78)	75.96 p = 0.46	68.08 (16.18)	72.83 p = 0.29
	Coping	42.65 (24.70)	55.60 **p < 0.0001**	38.71 (20.89)	59.11 **p < 0.0001**	50.37 (22.13)	54.60 p = 0.33	41.38 (26.80)	53.29 **p = 0.002**	42.68 (28.58)	56.06 p = 0.10
	Beliefs	68.84 (26.23)	69.13 p = 0.92	68.21 (23.99)	61.46 p = 0.11	68.06 (25.91)	67.80 p = 0.96	66.74 (28.95)	70.51 p = 0.33	80.36 (19.44)	78.09 p = 0.67
	Treatment	48.88 (16.61)	55.12 **p = 0.0065**	44.29 (15.51)	63.66 **p < 0.0001**	48.81 (15.32)	45.49 p = 0.27	52.90 (17.49)	53.93 p = 0.66	44.39 (15.41)	53.56 **p = 0.044**
	Total (P + S + E)	74.27 (19.25)	73.10 p = 0.60	76.46 (15.04)	72.05 p = 0.09	70.29 (22.79)	66.15 p = 0.35	75.93 (20.10)	77.92 p = 0.46	69.81 (17.74)	74.59 p = 0.33

Values in bold are statistically significant

Also, we found the following significant differences in the four subgroups by gender and age: (1) Males aged 8–12 years: the physical score was higher than the reference score (81.67 vs 69.93; p = 0.0001), while the coping (38.71 vs 59.11; p < 0.0001) and the treatment score (44.29 vs 66.06; p < 0.0001) were lower; (2) females aged 8–12 years: the physical score was higher than the reference score (76.85 vs 69.93; p = 0.02); (3) males aged 13–18 years: the coping score was lower than the reference score (41.38 vs 53.29; p = 0.002); (4) females aged 13–18 years: the treatment score was lower than the reference score (44.39 vs 53.56; p = 0.044) (Table 3).

#### QoLISSY parent

The QoLISSY-Parent version was completed by 142 respondents. The mean scores for the three core domains were similar to those of their children: physical 78.40, social 71.68, emotional 68.57, with a total score of 72.38. The scores of the additional domains were: coping 42.98, beliefs 66.81, and treatment 48.29. The last two domains specific to QoLISSY parent version were future (79.44), and effect on parents (62.15). Compared to the reference values for parents provided in the QoLISSY User’s Manual [9], we found in our sample a significant higher score for the physical domain (78.40 vs 71.80; p = 0.0038), and a lower score for treatment (48.29 vs 55.18; p = 0.0023). When considering the reference values of the QoLISSY User’s Manual [9] for parents of GHD patients only (excluding parents of ISS patients), the comparisons were as follows: physical (78.40 vs 78.72; p = 0.87); social (71.68 vs 77.87; p = 0.0086); emotional (68.57 vs 74.37; p = 0.0072); coping (42.98 vs 44.14; p = 0.62); beliefs (66.81 vs 71.41; p = 0.11); treatment (48.29 vs 55.38; p = 0.0018); future (79.44 vs 83.04; p = 0.12); effects on parents (62.15 vs 72.97; p < 0.0001); total score (72.38 vs 76.98; p = 0.0218).

We also found the following significant differences in the six subgroups by gender and age: (1) Males aged 4–7 years: the treatment score was lower than the reference score (43.37 vs 60.53; p = 0.024); (2) Females aged 4–7 years: no significant differences; (3) Males aged 8–12 years: the physical score (78.21 vs 67.83; p = 0.002), the total score (73.93 vs 67.80; p = 0.039) and the future score (79.43 vs 69.62; p = 0.020) were higher than the reference score, while the treatment score (47.04 vs 55.71; p = 0.007) was lower; (4) Females aged 8–12 years: the social (71.30 vs 59.16; p = 0.042) and the future (82.22 vs 67.96; p = 0.006) scores were higher than the reference score; (5) Males aged 13–18 years: the treatment score was lower than the reference score (51.02 vs 65.95; p < 0.0001); (6) Females aged 13–18 years: the treatment score (45.28 vs 58.52; p = 0.006) and effects on parents score (57.47 vs 73.00; p = 0.032) were lower than the reference score (Table 4).

**Table 4:** Results of QoLISSY parent

Perspective	Domains	Total (n = 142)		Male 4–7 years (n = 7)		Female 4–7 years (n = 3)		Male 8–12 years (n = 35)		Female 8–12 years (n = 27)		Male 13–18 years (n = 56)		Female 13–18 years (n = 14)	
		Mean (SD)	Ref p	Mean (SD)	Ref p	Mean (SD)	Ref p	Mean (SD)	Ref p	Mean (SD)	Ref p	Mean (SD)	Ref p	Mean (SD)	Ref p
Parents	Physical	78.40 (20.80)	71.80 **p = 0.0038**	63.69 (26.54)	66.41 p = 0.80	80.56 (9.62)	68.48 p = 0.16	78.21 (18.75)	67.83 **p = 0.002**	74.54 (27.06)	64.21 p = 0.058	81.77 (17.92)	79.16 p = 0.28	79.76 (20.34)	77.93 p = 0.74
	Social	71.68 (24.61)	69.41 p = 0.37	62.05 (22.92)	66.47 p = 0.63	79.17 (19.09)	69.89 p = 0.49	73.48 (20.51)	66.93 p = 0.07	71.30 (29.42)	59.16 **p = 0.042**	73.21 (23.54)	75.59 p = 0.45	64.96 (31.06)	75.12 p = 0.24
	Emotional	68.57 (22.00)	68.50 p = 0.98	65.63 (25.32)	69.92 p = 0.67	77.08 (13.01)	76.39 p = 0.94	71.16 (17.94)	67.92 p = 0.29	65.86 (27.72)	63.16 p = 0.62	69.53 (21.71)	68.32 p = 0.68	63.17 (21.52)	70.87 p = 0.20
	Coping	42.98 (23.59)	45.07 p = 0.35	43.21 (17.18)	38.48 p = 0.49	35.83 (12.83)	47.54 p = 0.25	40.07 (17.44)	44.33 p = 0.16	46.57 (25.72)	44.24 p = 0.64	42.54 (25.38)	46.59 p = 0.24	46.43 (30.88)	47.73 p = 0.88
	Beliefs	66.81 (27.76)	67.62 p = 0.78	70.54 (32.62)	69.96 p = 0.96	77.08 (21.95)	77.50 p = 0.98	68.57 (24.51)	62.97 p = 0.19	62.27 (32.27)	66.78 p = 0.47	67.86 (26.47)	65.70 p = 0.54	62.95 (32.75)	72.29 p = 0.31
	Treatment	48.29 (18.15)	55.18 **p = 0.0023**	43.37 (15.07)	60.53 **p = 0.024**	52.38 (16.97)	45.18 p = 0.54	47.04 (17.94)	55.71 **p = 0.007**	46.63 (18.59)	48.15 p = 0.67	51.02 (19.44)	65.95 **p < 0.0001**	45.28 (14.95)	58.52 **p = 0.006**
	Future	79.44 (24.36)	74.85 p = 0.081	77.14 (31.87)	79.27 p = 0.87	81.67 (11.55)	86.47 p = 0.55	79.43 (23.76)	69.62 **p = 0.020**	82.22 (25.01)	67.96 **p = 0.006**	79.55 (23.16)	75.65 p = 0.21	74.29 (29.99)	80.79 p = 0.43
	Effect on parents	62.15 (24.24)	65.68 p = 0.15	44.81 (21.39)	62.01 p = 0.078	75.76 (11.21)	63.50 p = 0.20	59.09 (23.18)	57.90 p = 0.76	63.47 (24.57)	63.44 p = 1.00	66.03 (24.88)	70.98 p = 0.14	57.47 (24.26)	73.00 **p = 0.032**
	Total (P + S + E)	72.38 (20.51)	69.97 p = 0.27	63.80 (20.43)	67.60 p = 0.64	78.79 (9.19)	71.77 p = 0.32	73.93 (16.88)	67.80 **p = 0.039**	70.20 (26.59)	62.18 p = 0.13	74.21 (19.29)	74.36 p = 0.95	68.34 (22.95)	74.64 p = 0.32

Values in bold are statistically significant

## Discussion

To our knowledge, this is the first study that administered the EQ-5D questionnaire to Italian GHD children. In our study, the mean EQ-5D utility value was 0.95 (SD 0.09), while the mean EQ VAS score was 86.2 (SD 14.2). Although no reference data are available for healthy subjects under the age of 18, our values were not dissimilar to those of the study by Szende et al. [10] for the group of healthy subjects aged 18–24 with the mean EQ VAS (self-assessed health) score of 87.5 [standard error (SE) 0.06], and the mean utility value of 0.985 (SE 0.003). However, in our study the proportion of respondents who indicated problems in the 5 dimensions of the EQ-5D was higher than that of healthy individuals aged 18–24 in the reference group reported by Szende et al. [10]: mobility: 7.7% vs 1.0%; self-care: 5.6% vs 0.7%; usual activities: 9.9% vs 1.8%; pain/discomfort: 16.9% vs 8.6%; anxiety/depression: 11.3% vs 5.2%. We note that the comparison group of healthy subjects aged 18–24 should be considered a proxy, and could differ to our sample in some respects, in particular compared to the younger boys and girls. Thus, the comparison with our results should be interpreted with caution.

Regarding subgroup analyses, we found that 4–7 years old boys were more likely to have some problems in self-care and usual activities than older children or adolescents. Nevertheless, these differences could be also due to the limited sample size of this age group, including only 7 boys. In our study, females aged 13–18, were more likely to have problems than males of the same age in the mobility (14% vs 2%), self-care (7% vs 0%), and anxiety or depression (29% vs 7%). However, the results also in this case should be interpreted with caution given the limited number of 14 females. Basically, the overall generic HRQoL of our study population can be considered high, and in line with the international reference values.

As for disease-specific HRQoL measured in children and adolescents through the QoLISSY-child version, in the overall study population we found a significantly higher score than the reference value for the physical domain (78.66 vs 73.69), and lower scores for coping (42.65 vs 55.60) and treatment (48.88 vs 55.12). Instead, when we compared our results to the reference values specific for GHD patients (not considering ISS patients), our mean scores were significantly lower for all domains, except physical.

As for disease-specific HRQoL measured in parents through the QoLISSY-parent version, in the overall study population we found a significantly higher score than the reference value for the physical domain (78.40 vs 71.80), and a lower score for treatment (48.29 vs 55.18). Comparing our results with those of parents of GHD patients only (excluding parents of ISS patients), our mean scores were significantly lower for social, emotional, treatment, effect on parents, and total score domains.

### Comparison of our results with those of other Italian or international studies

Comparing our results with those from a field test which describes the validation of the QoLISSY in Italy [7], we did not find significant differences. In the QoLISSY-child version (sample: 24 children) our means were: not significantly higher for physical (78.66 vs 70.83 of the QoLISSY Italian validation study; p = 0.07), social (74.10 vs 67.06, p = 0.17), emotional (71.14 vs 63.80; p = 0.09), beliefs (68.84 vs 63.28; p = 0.33), and total score (74.27 vs 67.23; p = 0.09); they were very similar for coping (42.65 vs 43.26; p = 0.91) and treatment (48.88 vs 48.66; p = 0.96). In the parent version (sample: 32 parents), our means were: not significantly higher for social (71.68 vs 67.83; p = 0.41), beliefs (66.81 vs 63.54; p = 0.55), effects on parent (62.15 vs 58.21; p = 0.40); very similar for physical (78.40 vs 78.78; p = 0.92), emotional (68.57 vs 67.22; p = 0.75), future (79.44 vs 78.39; p = 0.82) and total score (72.38 vs 71.36; p = 0.80); and not significantly lower for coping (42.98 vs 46.70; p = 0.42).

The systematic review by Backeljauw et al. [4] included five studies [11–15] comparing QoL between children with short stature due to GHD or ISS and children with normal stature; four of these studies [12–15] reported evidence of lower scores in children with short stature than normal stature children, while one study [11] found no difference. None of these studies used the EQ-5D or the QoLISSY questionnaires.

The review included also nine studies comparing QoL between short stature subgroups [14, 16–23]. Five out of 9 studies [14, 16, 19, 20, 23] did not find significant differences in QoL based on different causes of short stature or treatment status; considering only those studies using QoLISSY in GHD children, Bloemeke et al. [16] reported a mean QoLISSY total score after 12 months of treatment of 61.60 (SD 22.88), that is lower than the mean total score of our study (72.38), while Quitmann et al. [19] reported a mean QoLISSY total score after 12 months of treatment of 53.61 (SD 24.39), also in this case lower than the mean total score of our study.

Two out of nine studies [17, 18] found that QoL was significantly higher in children with less severe short stature than in children with more severe short stature. Bullinger et al. [17] reported a mean QoLISSY total score in GHD or ISS children of 73.10 (SD 21.39), similar to our mean total score (72.38). Drosatou et al. [18] reported a mean QoLISSY total score in GHD or ISS children according to the short stature level: height SDS ≤ − 2.0, 75.37 (SD 13.45); height SDS > − 2.0, 79.81 (SD 13.27). The first score (SDS ≤ − 2.0) was similar to our result.

Sommer et al. [22] reported that children with ISS had better QoL than SGA children with short stature. The study by Silva et al. [21] did not find differences between ISS and GHD children, while the treated children had significantly better HRQoL on the QoLISSY-child—physical domain compared to untreated ones; the mean QoLISSY physical score at baseline was 80.91 (20.16), similar to our mean physical score (78.40).

The only Italian study included in the review of Backeljauw [4] was the conference proceeding by Bettini et al. [5]. This study used the QoLISSY questionnaires (child and parent versions) in 80 GHD children and their parents, reporting a total QoL (defined as “satisfying QoL score”) for children of 85.7%, and of 60% for parents; however, it is not clear how these percentages have been calculated and therefore we cannot compare these results with ours.

Two recent conference abstracts [24, 25] describe the quality of life of children treated with once-weekly LAGH compared to those treated with daily GH and that of their parents. The QoLISSY core module was administered to girls aged 3–11 years and boys aged 3–12 years, and to their parents in eight countries. After 12 months of treatment, the total score for QoLISSY-Child was 74.69 in the LAGH group vs 69.03 in the daiòy GH group. These scores were similar to that in our study (74.27); however, the results are not fully comparable due to the different population ages (3–11/12 vs 4–18 of our study). Parents showed lower total scores than their children (69.49 in the LAGH group vs 63.80 in the daily GH group).

## Strengths and limitations

This is one of the first studies assessing the quality of life of GHD treated children and adolescents and their parents in Italy; in particular, this is the first study that administered the EQ-5D questionnaire to Italian GHD children. Strengths of our study include that our sample size (n = 142 children and parents) is large enough to provide sufficient statistical power to our analyses, even compared to most other national and international studies. To evaluate HRQoL we used two validated questionnaires, one generic and one disease-specific and we compared our results both with international reference values and with other national and international studies that administered the QoLISSY.

A potential limitation of this study is that our study population from north east and central Italy is under-represented compared to the Italian population, limiting the generalizability of the results to the whole country. Also, in some subgroups by age and gender there were few children and this could limit the statistical power of the comparisons. Another potential limitation is that we did not collect data on survey non-responding, so we could not calculate the response rate. Therefore, we were not able to compare the characteristics of respondents and non-respondents, in order to assess the potential non-response bias.

## Conclusion

Quality of life in patients with GHD, treated with GH, should be considered a critical outcome, along with improvement in height and other clinical endpoints. The results of this study suggest that the generic HRQoL in treated GHD patients is high, comparable to that of healthy people, while the disease-specific QoL in children and adolescents and their parents can be considered reasonably good, and comparable with that of international reference values of GHD/ISS patients. For children, our mean score was higher than the reference value for the physical domain and lower for coping and treatment subscales; for parents, we found a higher physical domain score, and a lower treatment score. When specific scores of patients with GHD are considered as reference values, most of our mean scores were lower than the international reference ones, for both the child and parent versions. On the other hand, comparing our results with those of the QoLISSY validation field test in Italy, we found no significant differences.

## References

[1] Hage C, Gan HW, Ibba A, Patti G, Dattani M, Loche S (2021). Advances in differential diagnosis and management of growth hormone deficiency in children. Nat Rev Endocrinol.

[2] Improda N, Capalbo D, Esposito A, Salerno M (2016). Muscle and skeletal health in children and adolescents with GH deficiency. Best Pract Res Clin Endocrinol Metab.

[3] Grimberg A, DiVall SA, Polychronakos C, Allen DB, Cohen LE, Quintos JB (2016). Guidelines for growth hormone and insulin-like growth factor-I treatment in children and adolescents: growth hormone deficiency, idiopathic short stature, and primary insulin-like growth factor-I deficiency. Horm Res Paediatr.

[4] Backeljauw P, Cappa M, Kiess W, Law L, Cookson C, Sert C (2021). Impact of short stature on quality of life: a systematic literature review. Growth Horm IGF Res.

[5] Bettini A, Teodori C, Maffei F, Ciofi D, Stagi S, editors (2019) The experience of pain in children with Growth Hormone Deficiency and psychosocial correlates: preliminary data from a longitudinal prospective study. In: Hormone Research in Paediatrics Conference: 58th Annual Meeting of the European Society for Paediatric Endocrinology: ESPE

[6] Orso M, Polistena B, Granato S, Novelli G, Di Virgilio R, La Torre D (2022). Pediatric growth hormone treatment in Italy: a systematic review of epidemiology, quality of life, treatment adherence, and economic impact. PLoS ONE.

[7] Quitmann J, Giammarco A, Maghnie M, Napoli F, Di Giovanni I, Carducci C (2017). Validation of the Italian Quality of Life in Short Stature Youth (QoLISSY) questionnaire. J Endocrinol Invest.

[8] Scalone L, Cortesi PA, Ciampichini R, Belisari A, D'Angiolella LS, Cesana G (2013). Italian population-based values of EQ-5D health states. Value Health.

[9] Chaplin J (2013) Quality of life in short stature youth: the QoLISSY Questionnnaire user's manual

[10] Szende A, Janssen B, Cabases J, editors (2014) Self-reported population health: an international perspective based on EQ-5D. Dordrecht (NL) 29787044

[11] Geisler A, Lass N, Reinsch N, Uysal Y, Singer V, Ravens-Sieberer U (2012). Quality of life in children and adolescents with growth hormone deficiency: association with growth hormone treatment. Horm Res Paediatr.

[12] Silva N, Bullinger M, Quitmann J, Ravens-Sieberer U, Rohenkohl A, Qo LG (2013). HRQoL of European children and adolescents with short stature as assessed with generic (KIDSCREEN) and chronic-generic (DISABKIDS) instruments. Expert Rev Pharmacoecon Outcomes Res.

[13] Stephen MD, Varni JW, Limbers CA, Yafi M, Heptulla RA, Renukuntla VS (2011). Health-related quality of life and cognitive functioning in pediatric short stature: comparison of growth-hormone-naïve, growth-hormone-treated, and healthy samples. Eur J Pediatr.

[14] Stheneur C, Sznajder M, Taylor M, Chevallier B (2011). Experience of adolescence in patients treated with GH during childhood. Pediatr Endocrinol Rev PER.

[15] Tanaka T, Tai S, Morisaki Y, Tachibana K, Kambayashi Y, Chihara K (2009). Evaluation of quality of life in children with GH deficiency and idiopathic short stature using the child behavior checklist. Clin Pediatr Endocrinol Case Rep Clin Investig.

[16] Bloemeke J, Silva N, Bullinger M, Witt S, Dörr HG, Quitmann J (2019). Psychometric properties of the quality of life in short statured youth (QoLISSY) questionnaire within the course of growth hormone treatment. Health Qual Life Outcomes.

[17] Bullinger M, Quitmann J, Power M, Herdman M, Mimoun E, DeBusk K (2013). Assessing the quality of life of health-referred children and adolescents with short stature: development and psychometric testing of the QoLISSY instrument. Health Qual Life Outcomes.

[18] Drosatou C, Vlachopapadopoulou EA, Bullinger M, Quitmann J, Silva N, Salemi G (2019). Validation of the Greek version of the quality of life in short stature youth (QoLISSY) questionnaire. J Pediatr Endocrinol Metab JPEM.

[19] Quitmann J, Bloemeke J, Dörr HG, Bullinger M, Witt S, Silva N (2019). First-year predictors of health-related quality of life changes in short-statured children treated with human growth hormone. J Endocrinol Invest.

[20] Shemesh-Iron M, Lazar L, Lebenthal Y, Nagelberg N, Tenenbaum A, Ezra R (2019). Growth hormone therapy and short stature-related distress: a randomized placebo-controlled trial. Clin Endocrinol.

[21] Silva N, Bullinger M, Sommer R, Rohenkohl A, Witt S, Quitmann J (2018). Children’s psychosocial functioning and parents' quality of life in paediatric short stature: the mediating role of caregiving stress. Clin Psychol Psychother.

[22] Sommer R, Blömeke J, Bullinger M, Quitmann J (2018). The psychometric evaluation of the quality of life in short stature youth (QoLISSY) instrument for German children born small for gestational age. J Endocrinol Invest.

[23] Tanaka T, Hasegawa T, Ozono K, Tanaka H, Kanzaki S, Yokoya S (2014). Effect of growth hormone treatment on quality of life in Japanese children with growth hormone deficiency: an analysis from a prospective observational study. Clin Pediatr Endocrinol Case Rep Clin Investig.

[24] Loftus J, Quitmann J, Valluri SR, Pastrak A, Reiter L, Roland CL (2020). PDB4 Evaluation of Quality of life in PRE-pubertal children using the quality of life in short stature youth (QOLISSY) questionnaire, following 12 months of growth hormone treatment with either a weekly somatrogon or a daily genotropin injection schedule. Value in Health.

[25] Loftus J, Quitmann J, Valluri S, Pastrak A, Reiter L, Roland C (2021). Comparison of quality of life responses from caregiver and children aged ≥ 7 years using the Quality of Life in Short Stature Youth (QoLISSY) Questionnaire, following 12 months of growth hormone treatment with either a weekly somatrogon or a daily genotropin injection schedule. J Endocr Soc.

